# Hibifolin, a Natural Sortase A Inhibitor, Attenuates the Pathogenicity of Staphylococcus aureus and Enhances the Antibacterial Activity of Cefotaxime

**DOI:** 10.1128/spectrum.00950-22

**Published:** 2022-08-01

**Authors:** Wu Song, Li Wang, Yinghua Zhao, Gongga Lanzi, Xingye Wang, Chi Zhang, Jiyu Guan, Wei Wang, Xuerui Guo, Ying Meng, Bingmei Wang, Yicheng Zhao

**Affiliations:** a College of Clinical Medicine, Changchun University of Chinese Medicine, Changchun, China; b Key Laboratory of Organ Regeneration and Transplantation of the Ministry of Education, Center for Pathogen Biology and Infectious Diseases, The First Hospital of Jilin University, Changchun, China; c Tibet University Medical College, Tibet, China; d Key Laboratory of Zoonosis, Ministry of Education, College of Veterinary Medicine, Jilin University, Changchun, China; e School of Pharmacy, Jilin University, Changchun, China; University of Calgary

**Keywords:** hibifolin, methicillin-resistant *Staphylococcus aureus*, sortase A, pneumonia, cefotaxime

## Abstract

This study aimed to identify hibifolin as a sortase A (SrtA) inhibitor and to determine whether it could attenuate the virulence of methicillin-resistant Staphylococcus aureus (MRSA). We employed a fluorescence resonance energy transfer (FRET) assay to screen a library of natural molecules to identify compounds that inhibit SrtA activity. Fluorescence quenching assay and molecular docking were performed to verify the direct binding interaction between SrtA and hibifolin. The pneumonia model was established using C57BL/6J mice by MRAS nasal administration for evaluating the effect of hibifolin on the pathogenicity of MRSA. Herein, we found that hibifolin was able to inhibit SrtA activity with an IC_50_ of 31.20 μg/mL. Further assays showed that the capacity of adhesion of bacteria to the host cells and biofilm formation was decreased in hibifolin-treated USA300. Results obtained from fluorescence quenching assay and molecular docking indicated that hibifolin was capable of targeting SrtA protein directly. This interaction was further confirmed by the finding that the inhibition activities of hibifolin on mutant SrtA were substantially reduced after mutating the binding sites (TRP-194, ALA-104, THR-180, ARG-197, ASN-114). The *in vivo* study showed that hibifolin in combination with cefotaxime protected mice from USA300 infection-induced pneumonia, which was more potent than cefotaxime alone, and no significant cytotoxicity of hibifolin was observed. Taken together, we identified that hibifolin attenuated the pathogenicity of S. aureus by directly targeting SrtA, which may be utilized in the future as adjuvant therapy for S. aureus infections.

**IMPORTANCE** We identified hibifolin as a sortase A (SrtA) inhibitor by screening the natural compounds library, which effectively inhibited the activity of SrtA with an IC_50_ value of 31.20 μg/mL. Hibifolin attenuated the pathogenic behavior of Staphylococcus aureus, including adhesion, invasion, and biofilm formation. Binding assays showed that hibifolin bound to SrtA protein directly. Hibifolin improved the survival of pneumonia induced by S. aureus USA300 in mice and alleviated the pathological damage. Moreover, hibifolin showed a synergistic antibacterial effect with cefotaxime in USA300-infected mice.

## INTRODUCTION

Staphylococcus aureus, a typical Gram-positive bacterium, is a dreadful pathogen infecting multiple tissues and organs in various vertebrate species, ranging from humans to livestock (1). Antibiotic resistance has long been a worldwide health problem due to the overuse and misuse of antibiotics (2). Methicillin-resistant Staphylococcus aureus (MRSA) and even vancomycin-resistant S. aureus (VRSA) have already threatened human health. MRSA has gained over 60% of all isolated S. aureus, and the incidence of MRAS has risen to 49% in the United States (3). Novel pathogen-specific therapeutic strategies for the prevention or treatment of serious bacterial infections have emerged, such as monoclonal antibodies (MAbs), nanomaterials, phages, and anti-virulence treatments (4–6). Therefore, innovative anti-infective agents are urgently needed to overcome the antibiotic resistance problem.

At present, the antivirulence treatment strategy has been an alternative approach that focuses on interfering with bacterial virulence factors instead of inhibiting bacterial growth or killing bacteria to treat infection (7). Virulence factors contain a variety of functions, including the direct effectors of bacterial pathogenicity and essential factors involved in host infection, host colonization, invasion of host tissues, adaptation to various host environments, destruction of host functions, and evasion of host defenses (8). Antivirulence strategy holds tremendous promise to be the next generation candidate for MRSA infection therapies.

Sortase A (SrtA) is a transpeptidase found in Gram-positive bacteria that can anchor surface proteins to the cell surface and recognize the C-terminal short peptide sequence LPXTG of the substrate protein, which forms an amide bond with the N-terminal glycine to the cell surface (9, 10). These surface proteins mediated by SrtA play an important role in bacterial adhesion, colonization, biofilm formation, and immune evasion (11, 12). Moreover, as a bacterial membrane enzyme, it is more likely to target intracellular proteins, making it an ideal anti-virulence target (13). The development of SrtA inhibitors has been an ideal strategy to combat antibiotic-resistant pathogen infections (14, 15).

Hibifolin, a flavonol glycoside, is isolated from the flowers of Abelmoschus manihot (Linn.) Medicus (16). The biological consequences and applications of hibifolin have been studied, including preventing Alzheimer’s disease (17), myocardial and cerebral ischemic injury (16), and possessing anti-inflammatory activities (18). In this study, we demonstrated that hibifolin attenuated the pathogenicity of S. aureus by directly targeting SrtA, which may be utilized in the future as adjuvant therapy for S. aureus infections.

Our previous study identified that the natural compound scutellarin has anti-SrtA activity (19). As a follow-up study, hibifolin was identified as a novel natural compound targeting SrtA by fluorescence resonant energy transfer (FRET) screening from our natural compound library. We determined that hibifolin disrupted the biological function of SrtA and displayed a protective effect against MRSA-induced lethal pneumonia, which may provide a new candidate for effective control of MRSA infection.

## RESULTS

### Hibifolin inhibited the activity of SrtA.

**FIG 1:**
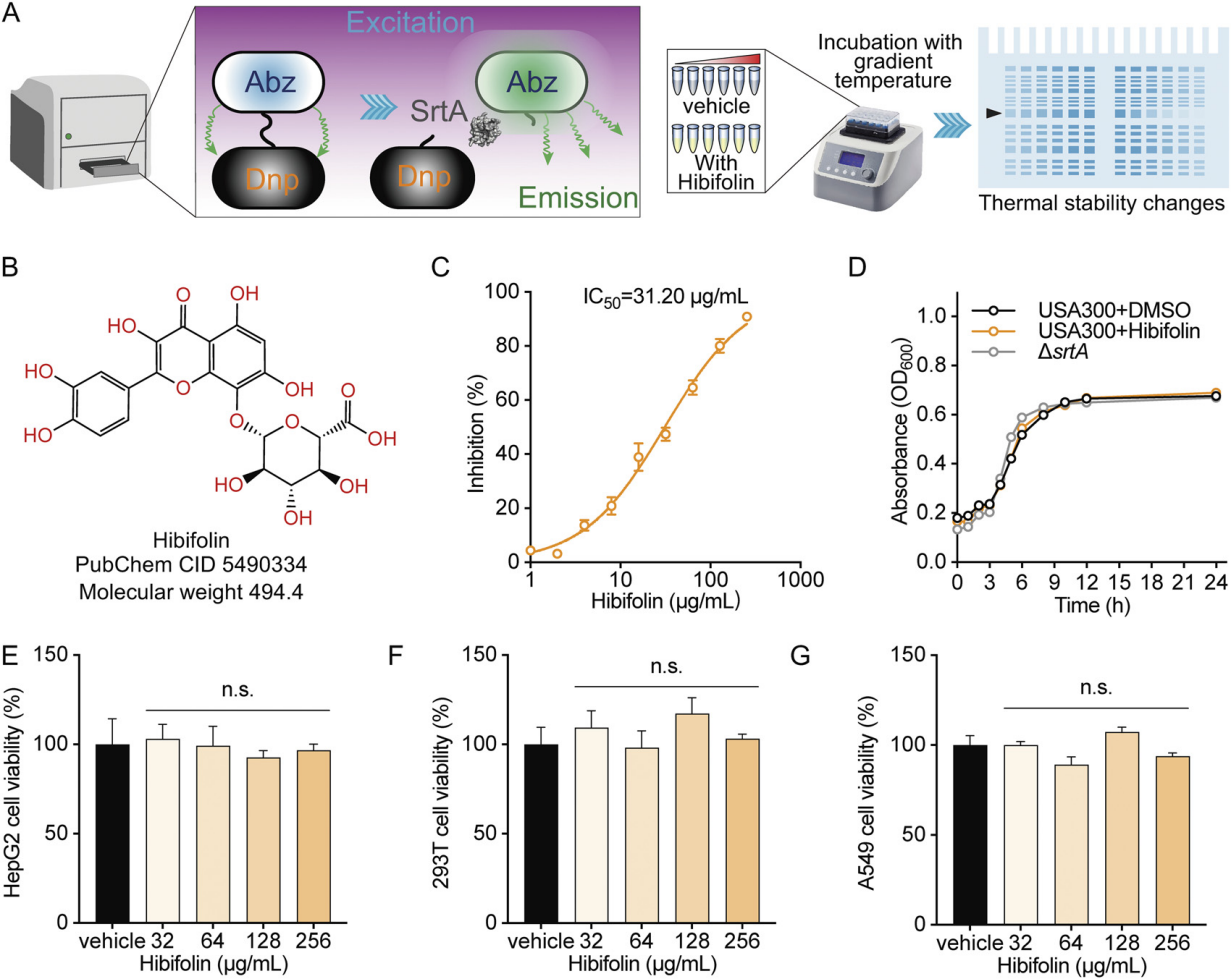
The inhibitory effect of the hibifolin on SrtA activity. (A) FRET and CETSA were used to screen inhibitors from natural small molecule compounds. (B) The chemical structure of hibifolin. (C) The effect of hibifolin on SrtA activity with IC_50_ of 31.20 μg/mL. (D) Growth curves of S. aureus USA300 with or without hibifolin treatment (256 μg/mL). The viability of HepG2 (E), HEK-293T (F), and A549 (G) cells viability measured by MTT assay after being exposed to various concentrations of hibifolin (0 to 256 μg/mL).

We first identified SrtA inhibitors from the natural compound using detection of the fluorescence intensity of the fluorescent substrate peptide Abz-LPATG-Dap (Dnp)-NH_2_ (Fig. 1A, left), which identified that the activity of hibifolin remarkably inhibited the activity of SrtA with an IC_50_ of 31.20 μg/mL (Fig. 1C). Subsequently, CETSA assays were further used to assess whether hibifolin was also an inhibitor of ClpP, MgrA or AgrAc and if there was a direct interaction between them (Fig. 1A, right). As shown in Fig. S2 in Supplemental File 1, the thermal stability of ClpP, MgrA, or AgrAc did not change with the increase in temperature, demonstrating that hibifolin was not an inhibitor of these three proteins. Next, we evaluated the antibacterial activity of hibifolin on S. aureus USA300. The MIC of hibifolin against USA300 was 512 μg/mL, along with the growth curve data (Fig. 1D), indicated that hibifolin barely influenced S. aureus USA300 growth. To assess the cytotoxicity, we performed MTT assays on A549, HepG2, and HEK-293T cell lines. As shown in Fig. 1E to G, there was no significant difference in cell viability among the groups, indicating hibifolin was almost noncytotoxic, at least to these cells. Hibifolin was able to inhibit the activity of SrtA and neither affected bacterial growth nor had cytotoxicity. These characteristics suggested that hibifolin has potential for development as a SrtA inhibitor.

### Hibifolin inhibited the adhesion of S. aureus to fibrinogen.

**FIG 2:**
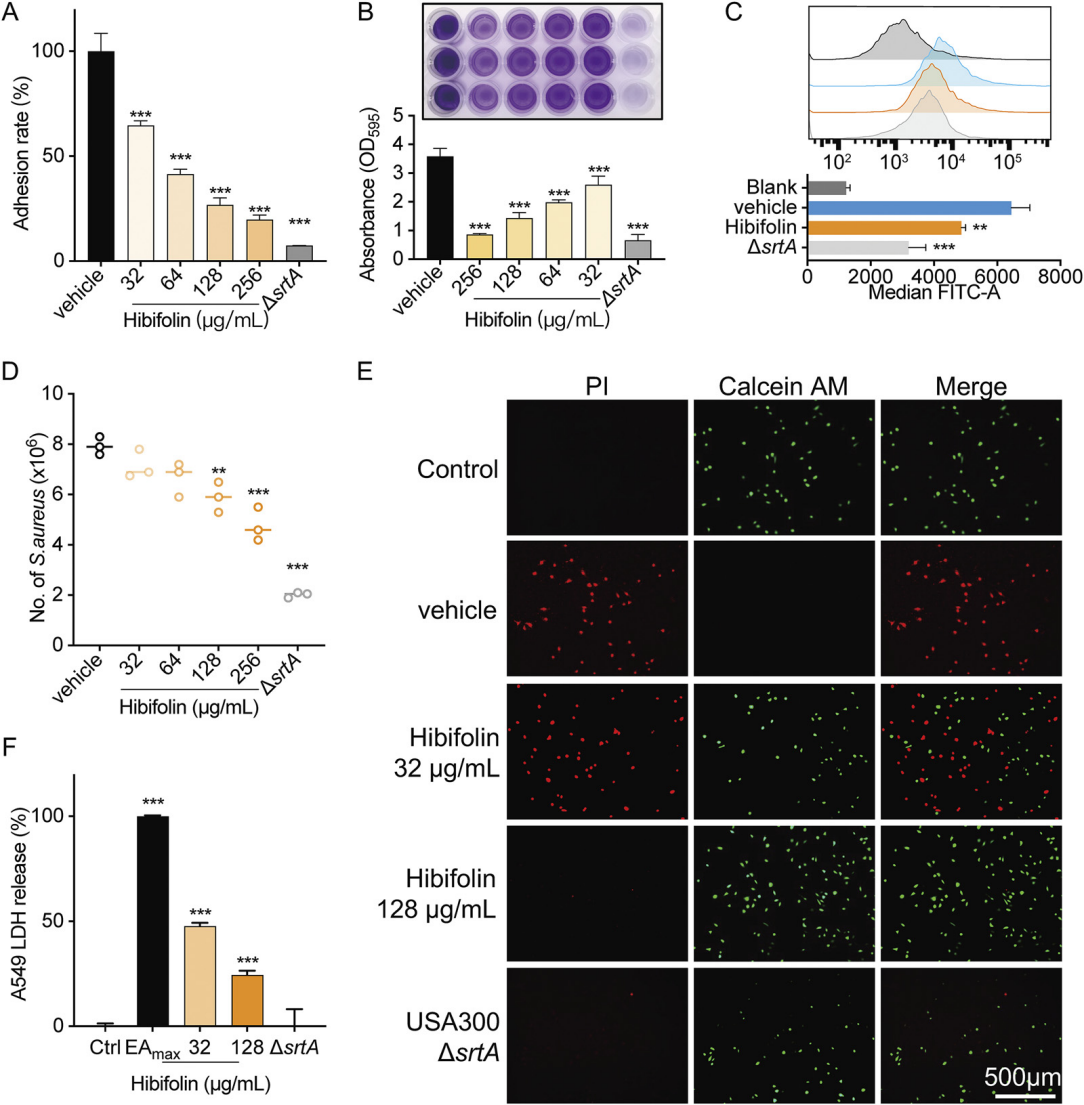
Effects of hibifolin on the sortase A-related virulence factors. (A) Multiple concentrations of hibifolin suppressed the adherence of S. aureus USA 300 to fibrinogen protein. (B) Biofilm formation is observed and quantified by staining with crystal violet. Hibifolin suppresses the S. aureus USA300 biofilm formation. (C) S. aureus surface protein (SpA) level is measured by flow cytometry using FITC-conjugated rabbit IgG. Δ*srtA* and USA300 represent a positive-control group and a negative-control group, respectively. (D) Different concentrations of hibifolin-treated S. aureus significantly inhibited bacterial invasion of A549 cells. (E) Live/dead staining confirmed that hibifolin reduced damage to A549 cells by S. aureus, and consistent results were obtained from LDH release assays (F).

The biological consequence of SrtA was evaluated under the intervention of hibifolin by the following experiments. An adhesion assay was performed to access the ability of hibifolin-treated S. aureus to adhere to fibrinogen. The result showed that the adhesion decreased to 26.85 ± 3.35% in the presence of 256 μg/mL of hibifolin compared to that in the USA300 group (Fig. 2A). The anchoring of surface proteins with LPXTG motifs to the cell wall was regulated by SrtA, leading to the formation of biofilms. We employed a crystal violet staining assay to assess biofilm formation. The results showed that hibifolin significantly inhibited biofilm formation, even at a low dose of 32 μg/mL, compared with that in the USA300 group (Fig. 2B). Surface protein A (SpA) served as a virulence factor that specifically combines with Fc region of IgG, thereby increasing the odds of immune evasion by interfering with opsonophagocytic clearance. To further detect the effect of hibifolin on the S. aureus USA300 surface protein, the fluorescence intensity of IgG labeling with FITC was assayed by flow cytometry to reflect the effect of hibifolin on the level of SpA. As shown in Fig. 2C, compared with the control group, the fluorescence was decreased in the hibifolin-treated group. In addition, when A549 cells were infected by S. aureus pretreated with hibifolin, the number of bacteria in the cells decreased significantly compared to the vehicle group (4.77 ± 0.67 versus 7.93 ± 0.36, *P* < 0.05), indicating that hibifolin could reduce the invasion of S. aureus on A549 cells (Fig. 2D). Live/dead staining and LDH release assays further revealed that the killing ability of hibifolin-treated S. aureus on A549 cells was significantly attenuated (Fig. 2E and F). These results demonstrated that hibifolin inhibited the SrtA-related virulence phenotypes, including adhesion, invasion, and biofilm-forming functions of MRSA *in vitro*.

### Hibifolin directly targeted SrtA.

**FIG 3:**
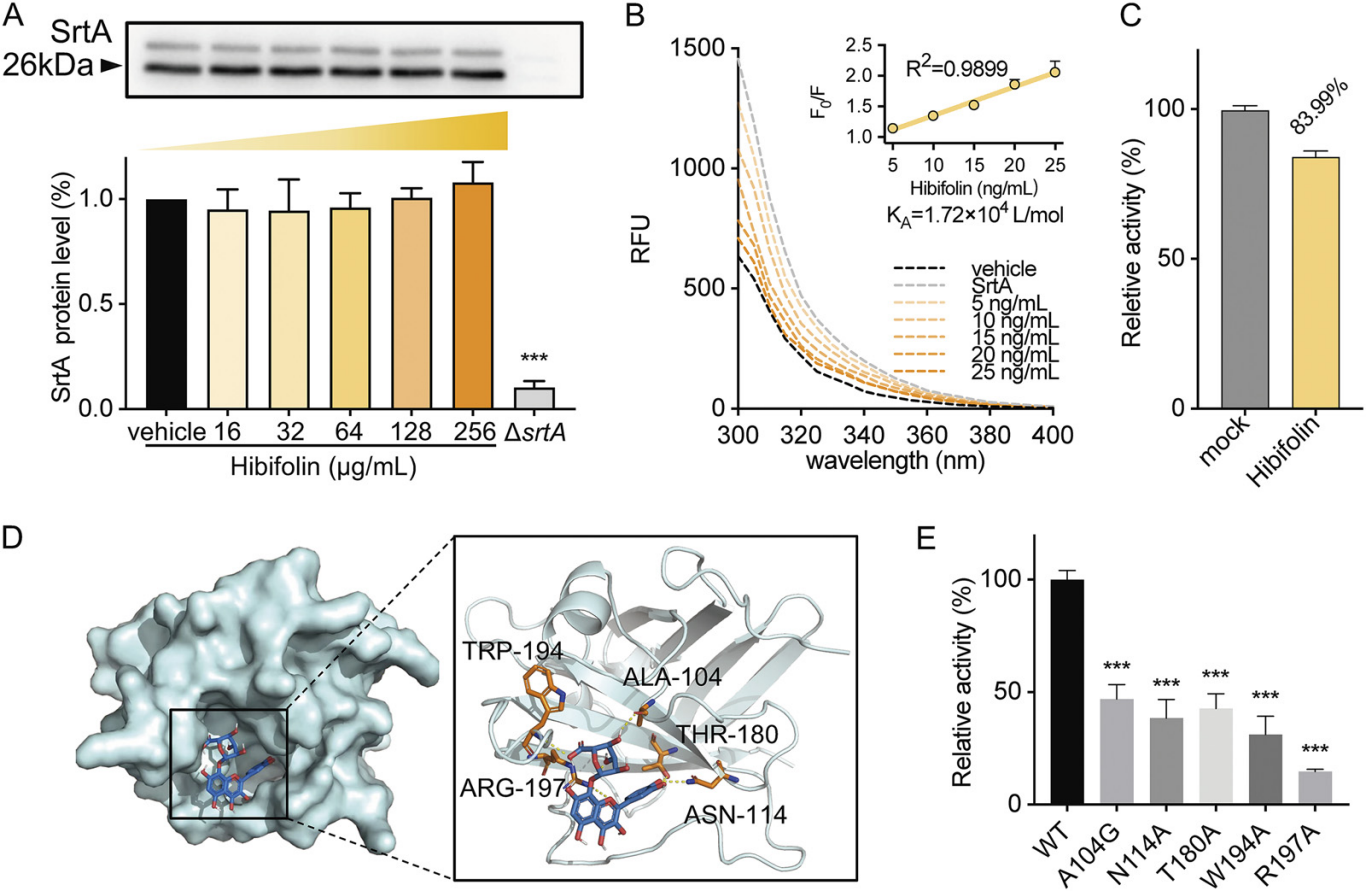
Determination of the binding sites for the hibifolin on SrtA. (A) Expression of different concentrations of hibifolin on SrtA expression by Western blot, which shows no significant effect. (B) The substrate-binding affinity of hibifolin was measured by SrtA fluorescence quenching. SrtA was treated with a 10-fold IC_50_ of hibifolin and then diluted the concentration. The group without hibifolin is assumed as 100% activity and the binding affinity is indicated by K_A_. (C) Reversible inhibitory effect of hibifolin on SrtA. (D) Molecular modeling predicted the interaction between hibifolin and SrtA. (E) Five mutants TRP-194, ALA-104, THR-180, ASN-114, and ARG-197 showed increasing resistance to hibifolin inhibition. Relative activities of recombinant SrtA were quantified via FRET assays.

To verify that hibifolin could directly bind to SrtA, Western blot and fluorescence quenching assays were carried out. The results showed that the expression of SrtA protein after treatment with different concentrations of hibifolin (0 to 256 μg/mL) was comparable to that of the untreated group, indicating that hibifolin did not affect the expression of SrtA (Fig. 3A). Fluorescence quenching analysis was employed to detect the binding affinity between hibifolin and SrtA. As shown in Fig. 3B, Hibifolin suppressed the fluorescence intensity of SrtA in a dose-dependent manner. We assessed the binding constants *K_A_* for hibifolin binding to SrtA by using fluorescence quenching experiments. The *K_A_* value was 1.72 × 10^4^ L/mol, revealing a direct binding interaction between SrtA and hibifolin.

To further analyze whether the inhibition of SrtA by hibifolin was reversible, SrtA was mixed with a 10-fold IC_50_ concentration of hibifolin and cultured with fluorescent peptide substrates. The recovery rate was 83.99 ± 2.06% compared with the control group (Fig. 3C), demonstrating that hibifolin acted as a reversible inhibitor of SrtA, which bonded noncovalently to the active site of SrtA.

Furthermore, molecular docking elucidated the binding mode between hibifolin and SrtA using Autodock vina 1.1.2 according to a previously documented procedure (20). The 3D structures were downloaded from the Protein Data Bank. We drew the 2D structure via the ChemBioDraw Ultra 14.0 (Fig. 3D). Discovery studio was used to analyze the interaction of docking. The molecular docking simulation indicated that hibifolin was bound to the binding pocket of SrtA, depending mostly on hydrogen bonding, and electrostatic interactions. In addition, it is well known that hydrogen bonding interactions play a crucial role in protein-ligand interaction and make an important contribution to the total binding affinity. In the present study, we analyzed the hydrogen bond networks and there were 5 hydrogen residues around the SrtA binding sides: THR-180, ASN-114, ALA-104, AGR-197, and TRP-194. Specifically, AGR-197 served as an important site in the SrtA active pocket while the side chain NH of AGR-197 formed two moderate frequency electrostatic and a hydrogen bond (Table S4 in Supplemental File 1). The molecular docking score was −6.8 kcal/mol. Following this lead, the activity of hibifolin on the mutated SrtA (TRP-194, ALA-104, THR-180, ARG-197, and ASN-114) was significantly lower than that of the wild-type (WT) group, and in particular, the ARG-197 mutant USA300 almost completely lost its activity, confirming the pivotal role of ARG-197 in the binding process of SrtA and hibifolin. In summary, these results verified the direct binding interaction between SrtA and hibifolin.

### Hibifolin reduced lung damage induced by S. aureus.

**FIG 4:**
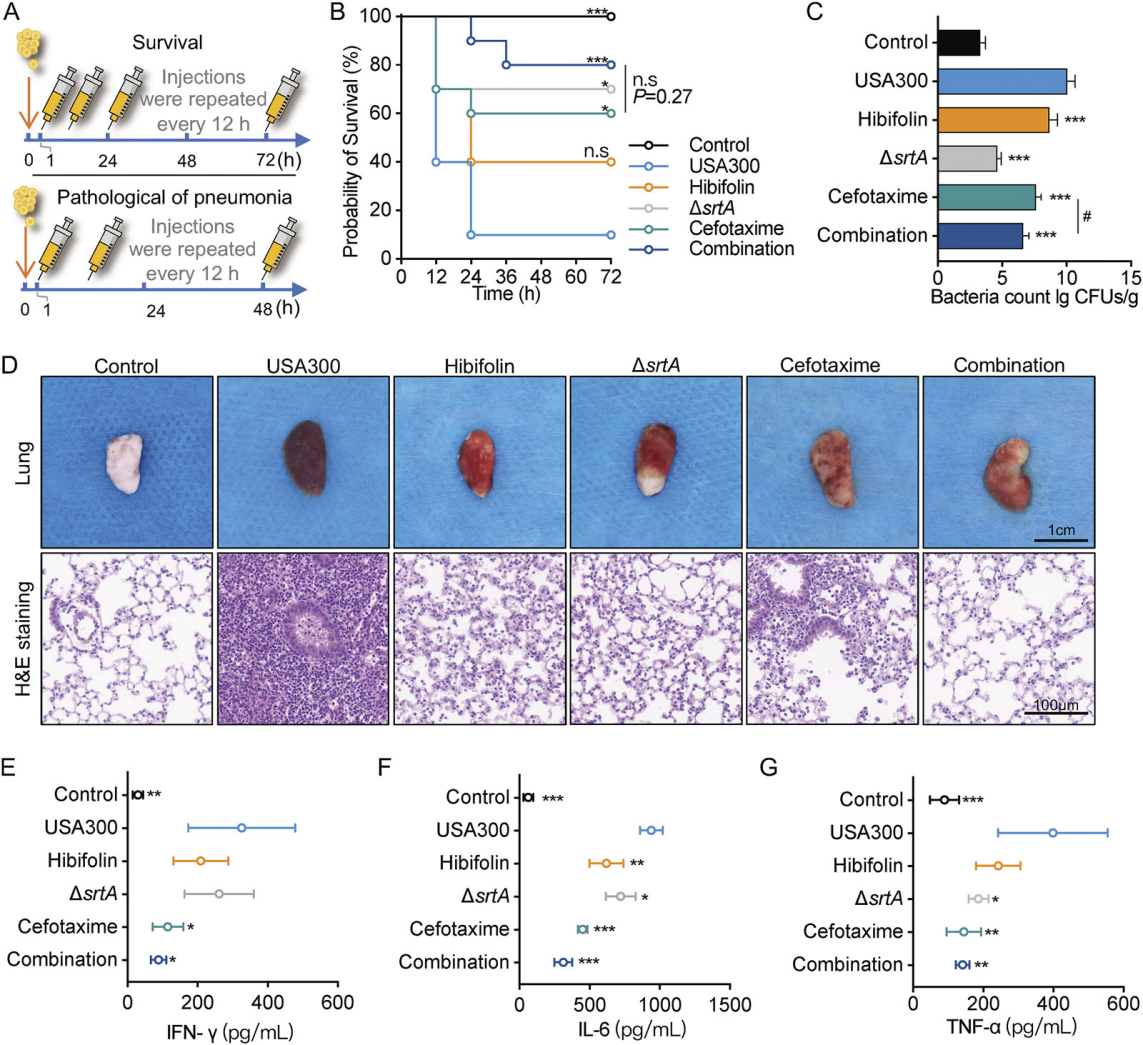
Protective effect of hibifolin and cefotaxime combination against lethal pneumonia in MRSA-infected mice. (A) Flow chart of MRSA-induced mouse pneumonia infection model. (B) Survival rate of C57BL/6J mice treated with hibifolin (100 mg/kg/d) or combination (hibifolin [100 mg/kg/d] + cefotaxime [40 mg/kg/d]) which infected with a lethal dose of S. aureus USA300. (C) CFU number in lung tissues. (D) Gross pathological changes in the lungs were observed by HE staining. Scale bar, 1 cm and 100 μm, respectively. (E–G) The levels of inflammatory cytokines (IFN-γ; IL-6; TNF-α) in the alveolar lavage fluid of each group of mice were determined by ELISA. **, *P* < 0.01; ***, *P* < 0.001 compared with the USA300 group; #, *P* < 0.05, comparisons were considered statistically significant.

The finding that hibifolin inhibited SrtA *in vitro* prompted us to evaluate the therapeutic effect of hibifolin *in vivo* against MRSA-induced pneumonia in mice. First, the checkerboard assays were used to screen for antibiotics synergistic with hibifolin. The results showed that the fractional inhibitory concentration index (FICI) of cefotaxime combined with hibifolin was 0.312 (FICI < 0.5 indicates synergy), which exhibited significant synergistic anti-MRSA effects *in vitro* (Table S2 in Supplemental File 1). Therefore, the therapeutic effect of the combination of hibifolin and cefotaxime on MRSA-induced pneumonia in mice could be further evaluated. In survival experiments, the survival rate of the infected mice was only 10% after 72 h of infection with S. aureus (2 × 10^8^ CFU), while the survival rate of the cefotaxime-treated group was 60%. The combination of hibifolin and cefotaxime increased survival by 20% compared to cefotaxime alone. There was no significant difference between cefotaxime treatment and the combination treatment (*P* = 0.27; Fig. 4B). In addition, compared with the infected mice group, the combination of hibifolin and cefotaxime markedly reduced the number of colonies in lung tissue. The difference between cefotaxime treatment and the combination treatment groups was statistically significant (*P* < 0.05, Fig. 4C). These data revealed that hibifolin in combination with cefotaxime protected mice from USA300 infection-induced pneumonia, which was more potent than cefotaxime alone. In terms of appearance and pathological changes, USA300-infected mice showed inelastic lungs with alveolar infiltration and severe damage observed under microscopy. In comparison, the lung tissues of the hibifolin in combination with the cefotaxime-treated group appeared to have a smooth, pinkish appearance with no obvious pathological changes. We then confirmed that hibifolin had a therapeutic effect on MRSA-infected pneumonia (Fig.4D). In addition, the levels of interferon (IFN)-γ, interleukin (IL)-6, and tumor necrosis factor (TNF)-α in the lung perfusate were significantly lower in the coadministered group (Fig. 4E to G). In conclusion, hibifolin reduced the virulence of S. aureus *in vivo* and *in vitro*, providing significant protection against pneumonia caused by S. aureus.

## DISCUSSION

Staphylococcus aureus is a prominent cause of infection in health care settings, with multidrug resistance to almost all known antibiotics (21). Therefore, to cope with the challenges brought about by the rapid emergence of drug resistance, developing novel anti-MRSA-infected therapy must be prioritized. Sortase A is essential for the pathogenicity of S. aureus. SrtA catalyzes the covalently binding of various surface virulence factor proteins to bacterial cell walls and plays a key role in the pathogenesis of Gram-positive bacteria, such as adhesion to host tissues, evasion of host defenses, and biofilm formation (12). Knockout of *srtA* inhibits the assembly and display of functional surface adhesins in the cell wall envelope and the ability to produce an acute infection (22). Therefore, interference with surface protein anchoring by targeting SrtA is a promising anti-virulence strategy to attenuate the pathogenicity of S. aureus.

At the present stage, available SrtA inhibitors include natural products, synthetic small molecule compounds, and designed peptide-analogs (23). Natural products provide an important source of SrtA inhibitors, and although this process is time-consuming, it may lead to the discovery of unexpected lead compounds. In addition, it has been suggested that the low incidence of infectious diseases in wild plants is because of the production of natural antimicrobial substances (24). These antimicrobial molecules have weak antimicrobial activity and may act in synergy with their production of natural anti-virulence substances that resist bacterial adhesion and invasion. For example, plants of the genus Berberis produce the low potency antibacterial substance berberine and 5′-methoxyhydnocarpin, of which 5′-methoxyhydnocarpin potentiates the antibacterial activity of berberine (25). This may partly explain the considerable amount of SrtA inhibitors in natural products.

Flavonoids are classified into 12 major subclasses based on chemical structures, such as anthocyanidins, flavanols, flavonols, flavones, flavanones, flavanonols, isoflavones, and others (26). In addition, each class of flavonoids can form glycosides (a genin with a flavonoid structure, containing a glycosidic moiety). The sugars can be joined to the flavonoid by tan O and by a C (among others), forming O-glycosides and C-glycosides (27). In our previous series of studies, we focused on SrtA and ClpP virulence targets. Until now, we have found that scutellarin (19) targets both SrtA and ClpP, quercetin (28), and myricetin (29) target ClpP. It is worth noting that although SrtA and ClpP have great differences in structure and biological functions, the inhibitors of these two targets screened from numerous natural compounds with different molecular structures belong to flavonoids. Although flavonoid inhibitors of these virulence targets have anti-infective effects, they also have their unique pharmacological effects, which provide more options for infected patients suffering from an underlying disease. What is noteworthy is that we have focused on the pan assay interference compounds (PAINS) (30), and we found that there was no PAINS group in hibifolin by searching the database (http://www.swissadme.ch/index.php). PAINS is usually a type of compound with a high hit rate that disturbs screening or detection methods. Its low potency is also an important factor influencing drug development.

With our accumulation of anti-virulence studies on many natural compounds, it appears that flavonoids and flavonol glycosides differ in their sensitivity to SrtA and ClpP. By listing a dozen of flavonoids and flavonol glycosides with their SrtA or ClpP relative inhibition rate, respectively (Table S5 in Supplemental File 1), we found that quercetin had the activity of inhibiting ClpP, while hibifolin, glycosylated quercetin, showed SrtA inhibiting activity. In addition, we enumerated two pairs of representative molecular structures that conform to glycosylation of flavonoids, “hibifolin-quercetin” and “scutellarein-scutellarin”. We focused on the following three indexes: inhibition rates of SrtA and ClpP, molecular docking data, and Gaussian data. First, the results of molecular docking in Fig. S3 in Supplemental File 1 showed that the flavonoid and the corresponding flavonoid glycoside both bind to the same site, the biding pocket, of SrtA or ClpP. Exceptionally, scutellarin prefers to bind the inactive site, which may also affect the polymerization of the ClpP protein which in turn affects its activity. From the Gaussian data and molecular data, we concluded that SrtA and ClpP affinity (docking score) increased with the increase of Gaussian ΔG, molecular volume, and steric hindrance energy after glycosylation, which seemed to imply that the glycosylation of flavonoids enhanced the affinity of flavonoids to both SrtA and ClpP (Table S6 in Supplemental File 1). The experimental data suggested that the second pair of data conforms to the rule we obtained from the virtual experiments above: glycosylation enhances the sensitivity to ClpP and SrtA. However, we did not find such a rule from the first pair. Although we have attempted to analyze the effect of glycosylation of flavonoids on the sensitivity of SrtA and ClpP, the evidence is far from adequate. We can only draw a vague conclusion from our existing data that glycosylation of flavonoids appears to enhance their sensitivity to ClpP and SrtA. As more evidence accumulates in the future, the structure-activity relationship will become clear.

**FIG 5:**
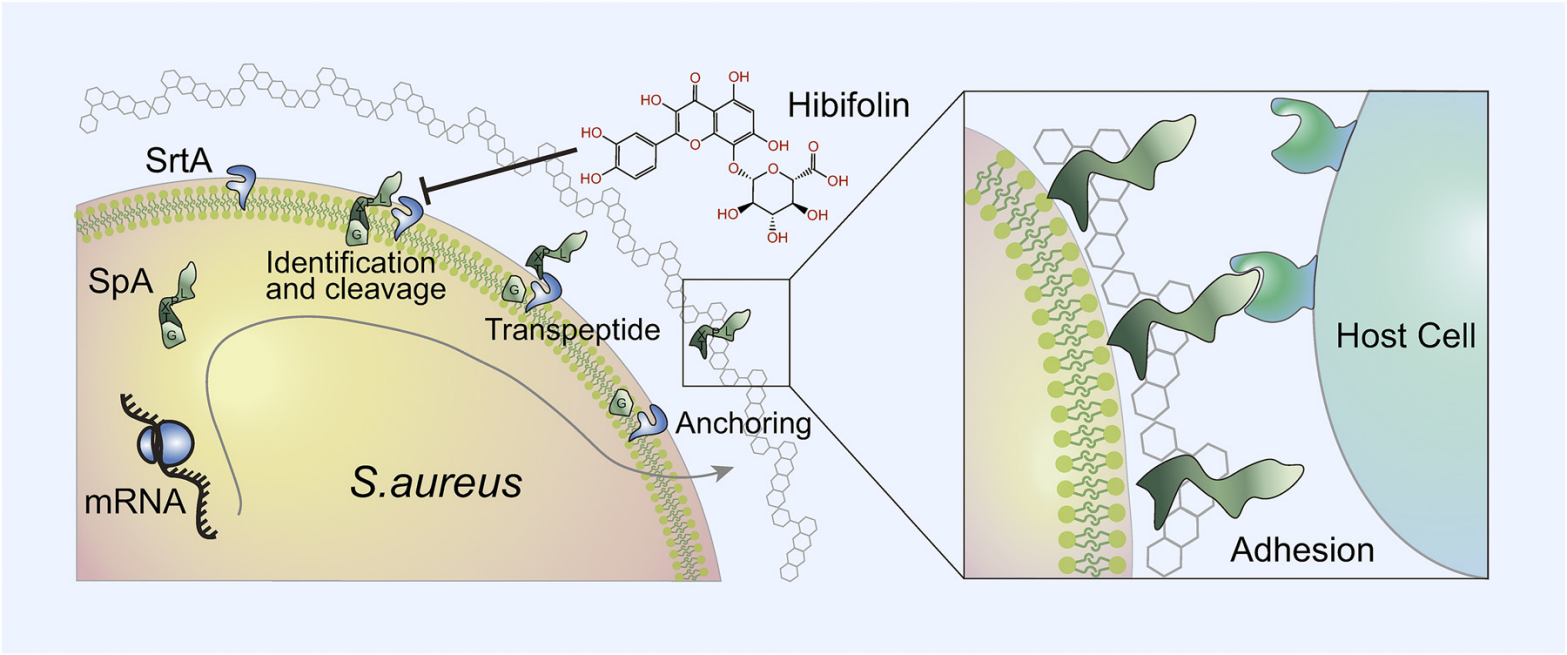
Hibifolin reduced the formation of biofilm of S. aureus and attenuated the adhesion and invasion to the host and the anchoring of surface proteins by inhibiting SrtA.

Studies have demonstrated that hibifolin has neuroprotective effects, as evidenced by protection against neuronal cell damage caused by amyloid β-protein as well as potential antidepressant effects (17). Our findings herein expand the anti-infective application of hibifolin. Natural compounds tend to be multitargeted compared to synthetic compounds by virtual screening. We, therefore, speculate that hibifolin may have multiple protective effects against severe S. aureus infections due to its pharmacological activity in addition to its anti-infective activity. Hibifolin reduced the virulence of S. aureus by inhibiting SrtA activity. We identified hibifolin from hundreds of natural compounds as a candidate capable of inhibiting SrtA activity with the IC_50_ of 31.20 μg/mL using an established screening method for SrtA inhibitors. We consider that hibifolin reduced the virulence of S. aureus by inhibiting SrtA activity, as SrtA is one of the most essential virulence factors in S. aureus. As shown in Fig. 5, hibifolin prevented SrtA from covalently anchoring surface proteins to the cell wall. Therefore, these surface proteins cannot play a role in bacterial adhesion and invasion of host tissues, biofilm formation, and immune evasion through inhibition and phagocytosis. Hibifolin visibly attenuated the virulence-related phenotype of SrtA by decreasing the adhesion of S. aureus to fibrinogen, reducing the ability of SpA displayed on the surface of the bacteria and biofilm formation. The results of fluorescence quenching and molecular docking revealed the interaction between SrtA and hibifolin. The *in vivo* results indicated that hibifolin could protect mice from lethal pneumonia brought on by MRSA.

The fluorescence intensity of fluorescence substrate Abz-LPETG-Dnp-NH_2_ was detected using FRET technology. FRET technology is a powerful tool for detecting nanometre-scale distances and changes in nanometre-scale distances between biological macromolecules *in vivo*, which is widely used to detect whether there is a direct interaction between two protein molecules in each cell (31). In Shen et al. (32) study, FRET technology was used to screen for compounds that promote the formation of A1R and D1R heterodimers. For another, the initial DNA melting screen was performed using a FRET DNA melting-based assay (33).

We agree with Cascioferro et al. (34), that the future of antivirulence agents should be in combination with antimicrobial or immune-activating drugs, which may not be strong enough on their own. In this study, we combined hibifolin with cefotaxime, the preferred first-line antibiotic for MRSA infection (35). Data from *in vivo* study showed that hibifolin exhibited a significant protective effect against USA300-induced lethal pneumonia, as evidenced by increased survival, improved pathological manifestations, and reduced levels of the inflammatory cytokine.

The available evidence is not enough to clarify the structure-activity relationship of flavonoid inhibition on virulence targets of S. aureus. We also look forward to the subsequent work on the structural optimization of hibifolin as a leading compound and the elucidation of the structure-activity relationship. In addition, there is a need for pharmacokinetic and safety information on hibifolin.

As a SrtA inhibitor of S. aureus, hibifolin significantly attenuates the pathogenicity of S. aureus and enhances the antibacterial activity of cefotaxime. Due to the properties of reducing the virulence and producing less selective pressure on MRSA, we should expect hibifolin to be applied as an adjuvant therapeutic agent for MRSA infections, which may help to reduce the use of antibiotics and delay the development of antibiotics resistance.

## MATERIALS AND METHODS

### Strains and reagents.

Staphylococcus aureus USA300 was purchased from ATCC (Manassas, VA, USA). Staphylococcus aureus USA300 SrtA deletion strain (Δ*srtA*) and pET28a-SrtA plasmid were established in our laboratory. Bovine fibrinogen was purchased from Yuanye Biology (Shanghai, China). A fluorescent substrate peptide Abz-LPATG-Dap (Dnp)-NH_2_ was purchased from LifeTein, LLC (Beijing, China). Hibifolin (purity > 98%) was purchased from Pufei De Biotech Co., Ltd. (Chengdu, China), and the graph of HPLC identification results is presented in Fig. S1 Conventional antibiotics were purchased from Sangon Biotech (Shanghai, China).

### Construction of SrtA and its mutant plasmids.

Construction of SrtA and its mutant plasmids was performed as in our previous work (19). The DNA sequence encoding the SrtA residues Gln-60-Lys-206 was amplified by PCR, using S. aureus USA300 genomic DNA as a template. The pET-28a vector was cloned after PCR products were digested in XhoI and BamHI. The pET-28a-SrtA Δ_Ν59_ plasmid was generated after sequence confirmation by DNA sequencing. Then, the Mut Express II Fast Mutagenesis kit V2 (Vazyme Biotech, Nanjing, China) was used for site-directed mutagenesis of TRP-194, ALA-104, THR-180, ARG-197, and ASN-114. The mutant primer pairs used to create the mutants are shown in Table S1 in Supplemental File 1.

### Expression and purification of SrtA and its mutant protein.

The recombinant pET28a-SrtA plasmid and its mutant plasmids were transferred into E. coli expression host BL21(DE_3_) by heat shock method, respectively (36). The bacteria were then cultured until the optical density at 600 nm (OD_600_) reached 0.8 and continued to culture with 0.5 mM IPTG at 16°C for 8 h. Based on the recombinant pET28a-SrtA plasmid containing 6 × His tags, therefore the induced protein was purified by Ni-NTA (Beyotime Biotechnology, Shanghai, China). Subsequently, the nonspecific protein was eluted with 10 mM imidazole, and then the purified protein was eluted with 100 mM imidazole.

### SrtA inhibitor screening.

Fluorescence resonant energy transfer (FRET) was used to screen the effect of the SrtA inhibitor (22). Purified SrtA (4 μM) and hibifolin with different concentrations were added into a 100 μL reaction system, incubating at 37°C for 1 h. A fluorescent substrate peptide Abz-LPATG-Dap (Dnp)-NH_2_ (10 μM) (Beijing, China) was added to initiate the reaction. After incubating at 37°C for 20 min, the fluorescent microplate reader was measured with the excitation wavelength at 309 nm and the emission wavelength at 420 nm. Subsequently, a gradient concentration inhibition curve was performed and IC_50_ was calculated. When the inhibitory activity was greater than 60%, the compound was considered a potential inhibitor. The representative list of our nature compounds library has been compiled in Table S3 in Supplemental File 1 along with the results of the initial screening.

### Reversible inhibition experiment of SrtA.

A reversible assay was performed in accordance with the previous study (19). Ten-fold IC_50_ of hibifolin was added to 100 μL of the purified SrtA (4 μM) at 37°C for 1 h. Then, the above reaction solution was diluted at 1:100. Subsequently, the substrate peptide (10 μM) was added and incubated at 37°C for 20 min. The fluorescence intensity was read under the excitation wavelength was 309 nm, and the emission wavelength was 420 nm.

### Growth assays.

Growth assays were carried out with reference to a previous literature report (37). The overnight cultured S. aureus USA300 was diluted at 1:100 and transferred into fresh BHI broth with or without 128 μg/mL hibifolin, culturing until the OD_600_ was 0.3. The bacterial solution was divided into the control group (WT +DMSO), drug group (WT + hibifolin), and Δ*srtA* group. The growth was recorded at 1 h intervals within 24 h. A UV spectrophotometer measured the growth curve at an absorbance of 600 nm.

### Checkerboard studies.

The checkerboard microtiter assay (38) was applied for determining the interaction of combinations of hibifolin with Cefotaxime, Doxycycline, Ceftriaxone Sodium, Vancomycin, Oxacillin sodium, Ceftiofur Sodium, Cefepime, Ceftaroline fosamil, relatively. One hundred microliters of CAMHB medium were added to a 96-well plate, followed by a fold dilution of hibifolin and antibiotics. MRSA USA300 (10^5^ CFU/well) was added to each well and the MIC was calculated as wells with the lowest concentrations of drugs with no visible growth after incubation at 37°C for 16 h. The fractional inhibitory concentration index (FICI) of each combination were calculated according to the following formula: FICI = FIC_antibiotic_ + FIC_hibifolin_ = MIC_combine-antibiotic_/MIC_antibiotic_ + MIC_combine-hibifolin_/MIC_hibifolin_. FICI ≤ 0.5 indicates synergism; 0.5 < FICI ≤ 1 indicates additivity; 1 < FICI ≤ 2 indicates irrelevance; FICI > 2 indicates antagonism.

### Cell viability.

Cell viability was carried out as previously described (39). Human hepatocellular carcinoma (HepG2), human kidney epithelial cell line (293T), and human non-small cell lung cancer (A549) cells of 2 × 10^4^ cells/well were seeded in 96-well plates for 24 h with 5% CO_2_, relatively. Different concentrations of hibifolin (0 to 256 μg/mL) were then added and maintained at 37°C for 24 h. Then, 0.5 mg/mL of MTT solution was incubated in 96-well plates after discarding the medium. The plates were set in the cell incubator for 4 h and the crystals were dissolved with DMSO. The absorption at 490 nm was measured with a microplate reader.

### Fibronectin-binding assay.

A fibronectin-binding assay was completed as described but with slight revision (40). Bovine blood fibrinogen (Fg) was added into 96-well plates at 20 μL/mL per well followed by cultured overnight at 4°C. After being washed by PBS, 100 μL of 1% BSA was added into each well of 96-well plates and blocked the medium for 2 h. The cultures of S. aureus USA300 in the BHI medium and placed on a shaker at 200 rpm for 24 h, and different concentrations of hibifolin were added, among which the USA300, Δ*srtA,* and BHI medium represented negative, positive, and control groups. The bacterial culture was cultured at 37°C until OD_600_ = 0.5. After incubating, the unbound bacteria were gently washed using PBS and fixed with 4% formaldehyde for 30 min. After dying with crystal violet dye for 20 min, the absorbance at 595 nm was measured by a microplate reader.

### Biofilm formation.

The 96-well plates were added 100 μL of 20% freeze-dried rabbit blood at 4°C for 24 h. Staphylococcus aureus USA300 or Δ*srtA* were diluted at a ratio of 1:100 and cultured in BHI broth supplementing with 0.5% glucose and 3% NaCl. Then, the solution in 96-well plates was carefully discarded, and 100 μL bacterial solution per well with different concentrations of hibifolin (32 to 256 μg/mL) was added to each well, respectively. The 96-well plates were cultured at 37°C for 24 h, then cell plates were washed three times with PBS to remove unbound bacteria. Then biofilm was evaluated by 0.1% crystal violet staining for 20 min (41). Gently rinsed unbound dyes with sterile PBS buffer and blown dried at room temperature. After that, 200 μL of 95% ethanol was added to each well. Relative biofilm formation was determined by measuring the absorbance at 570 nm.

### Surface protein analysis.

The overnight cultures of S. aureus USA300 and Δ*srtA* were diluted 1:100 in BHI medium. The cultures were then incubated on a shaker to an OD_600_ of 0.3. Hibifolin was added with different concentrations until the OD_600_ was 1.0. The bacteria were collected at 5,000 rpm for 5 min and washed with PBS twice a time. After incubating with 0.5% BSA for 20 min, and rewashing with PBS, the USA300 were resuspended with 4% formaldehyde and collected with centrifugation (40). The USA300 were resuspended with 50 μL FITC labeled rabbit IgG (1:100) and incubated in the dark at 37°C for 2 h. After washing twice to remove the dye, the bacteria were resuspended with PBS. The fluorescence intensity was measured by flow cytometry (Beckman Coulter, USA) to evaluate the amount of SpA.

### Cell invasion assays.

The invasion of A549 cells by S. aureus was detected as previously described (40). A549 cells were incubated in 24-well plates at 2 × 10^4^ per well overnight (37°C, 5% CO_2_). Overnight cultured S. aureus USA300 and Δ*srtA* were diluted 1:100 into a 1 mL system and hibifolin with a final concentration of 256,128,64,32 μg/mL was added. Then the bacteria solution was incubated until the OD_600_ reached 1.0 with a shaker rate of 200 rpm at 37°C. USA300 and Δ*srtA* were set as negative and positive controls. Then, 400 μL bacterial solution was added to 24-well plates and cultured in a 37°C biochemical incubator for 2 h. After each well was washed three times with PBS, 1 mL of DMEM supplemented with 300 μg/mL gentamicin was added and incubated for 1 h at 37°C. The A549 cells on the coverslips were then lysed with 1% Triton X-100 for 30 min and plated onto BHI broth agar plates for CFU.

### Live/dead assays.

The analysis of live/dead assays was performed using a previously described method (42). A549 cells were cultured in DMEM, with 10% fetal bovine serum (FBS). After being digested with 0.25% trypsin, the cells were seeded in 24-well plates at 1 × 10^5^ per well and cultured at 37°C, 5% CO_2_ for 24 h. Next, fresh DMEM with S. aureus and different concentrations of hibifolin (32 and 128 μg/mL) was used to replace the culture medium and was incubated for 5 h at 37°C. Then the Live/Dead (green/red) reagent (Beyotime, Shanghai, China) was used to assess the therapeutic effect of hibifolin on A549 cells using a confocal laser scanning microscope.

### Cellular thermal shift assay (CETSA).

E. coli BL21(DE3) containing pET28a-ClpP, MgrA, or AgrAc was used to induce the relative expression of protein by adding 0.5 mM IPTG, and the supernatant of the lysate was collected by centrifugation after ultrasonic fragmentation (43). The supernatant was incubated with hibifolin at 37°C for 1 h through centrifugation at 18,000 g for 20 min at 4°C. The samples were heated to various temperatures at a temperature gradient for 5 min, respectively. We then put samples into ice water for 3 min immediately and gently centrifuged at 18,000g for 20 min to yield a supernatant. After being analyzed by SDS-PAGE, the samples were incubated with Coomassie brilliant blue G-250 stain, and the relative intensities of the indicated proteins were visualized using ImageJ software.

### Western blot.

To assess the effect of hibifolin on SrtA expression in S. aureus USA300, different concentrations of hibifolin (0 to 128 μg/mL) were added to S. aureus USA300 medium, shaking at 220 rpm at 37°C until the OD_600_ value reached 2.0. The total proteins of S. aureus were isolated according to the conventional method (44). The total proteins were separated by 12% SDS-PAGE, transferred to polyvinylidene fluoride (PVDF) membrane, blocked with 5% BSA for 2 h, and incubated with rabbit anti-SrtA polyclonal antibody (1:3000, prepared in the laboratory) at room temperature. Following incubation with HRP-labeled goat anti-rabbit IgG (1:10000) for 1 h at room temperature, the membrane was visualized using a super ECL Plus (Beyotime, Beijing China) and the gray value of the target band was analyzed using ImageJ software.

### Fluorescence quenching.

The binding constants of hibifolin with SrtA and the SrtA mutant were measured by fluorescence quenching assay as previously described (40). The SrtA fluorescence emission spectra were recorded in the absence or presence of different concentrations of hibifolin. The excitation wavelength was set to 280 nm using a fluorescence spectrophotometer, and the emission spikes were recorded at 280 to 400 nm. The widths of the excitation and emission slits were 5 and 10 nm, depending on the width of the slit. The relative fluorescence intensity was plotted against different concentrations of hibifolin based on fluorescence quenching to obtain data and *K_A_* values were calculated.

### Molecular docking.

Although the X-ray crystallography 3D structure (PDB accession no. 1T2P) was in Protein Data Bank, the structure of SrtA was obtained. The 3D structures of hibifolin were constructed by the ChemBio3D Ultra 12.0 software package. A standard docking procedure for a SrtA protein and hibifolin was performed with AutoDock-Tools 1a.5.6 software. Finally, the discovery studio was used to analyze the interaction of docking.

### Murine pneumonia models.

To investigate the therapeutic effect of hibifolin on acute pneumonia caused by MRSA, the murine pneumonia models were established using C57BL/6J mice (18 to 22 g, 6 to 8 weeks old) (45). Mice were infected intranasally with 2 × 10^8^ CFU of S. aureus USA300 suspension and then held for 30 s to guarantee that each mouse would inhale bacteria into its lungs. After 1 h of infection, mice were injected subcutaneously with hibifolin (100 mg/kg/d) or combination (hibifolin [100 mg/kg/d] + cefotaxime [40 mg/kg/d]). They were then reinjected in 12 h intervals, and the survival rate of mice was recorded every 12 h for 96 h.

To further assess the therapeutic effect of hibifolin on pneumonia in mice, the bacterial load in the lung tissues and histopathological changes in the lungs were analyzed. Mice in each group were allowed to remain infected for up to 2 days by nasal drip injection of 30 μL (1 × 10^8^ CFU) of S. aureus culture. Then, mice were sacrificed using the cervical dislocation method, and the lungs were collected, weighed, and homogenized. The homogenate was then adequately diluted and spread on BHI agar plates. After the plate was incubated overnight at 37°C, the number of colonies was counted. The left lung of each group was collected, perfused, and fixed in 10% formalin. The pathological changes in the lung tissue were observed under a light microscope after staining with routine hematoxylin and eosin (H&E). In addition, alveolar lavage fluid was collected from each group of mice and changes in inflammatory factors (IFN-γ, IL-6, and TNF-α) were measured by ELISA kit (Sbjbio, Nanjing, China).

### Statistical analysis.

The statistical significance of the treated and USA300 group was assessed using the log-rank tests for the survival curves (**, *P* < 0.01; ***, *P* < 0.001, compared with the USA300 group). For other assays, a Student's *t* test was used. *P* < 0.05 was considered statistically significant. The differences were analyzed using GraphPad Prism 8.0 statistical software. The data are presented as the mean ± SD.
