# Supplementary material for: Hibifolin, a Natural Sortase A Inhibitor, Attenuates the Pathogenicity of Staphylococcus aureus and Enhances the Antibacterial Activity of Cefotaxime
Source: Microbiol Spectr. 2022 Aug 1;10(4):e00950-22. doi: 10.1128/spectrum.00950-22 (PMC9430695; doi:10.1128/spectrum.00950-22)
Supplement: Supplemental file 1 — Supplemental material. Download spectrum.00950-22-s0001.pdf, PDF file, 1.4 MB [file spectrum.00950-22-s0001.pdf]

Supplementary Information to

Hibifolin, a natural sortase A inhibitor, attenuates the pathogenicity of *Staphylococcus aureus* and enhances the antibacterial activity of cefotaxime

Wu Song, Bingmei Wang, Li Wang, Xingye Wang, Xuerui Guo, Xiangri Kong, Yanhe Luan, Jiyu Guan, Yan Shi, Tiedong Wang, Yicheng Zhao

To whom Correspondence should be addressed:

yichengzhao@live.cn

Table of contents

Supplementary Figure.1 Quality test report of hibifolin.

Supplementary Figure.2 CETSA results of purified proteins with hibifolin.

Supplementary Figure.3 Interaction studies between two pairs of flavonoid and flavonoid glucosides compound with SrtA and ClpP proteins.

Supplementary Table.1 Primers used in article.

Supplementary Table.2 Antimicrobial effects of antibiotics in combination with hibifolin in *S. aureus*.

Supplementary Table.3 Compounds library for screening SrtA inhibitors in this study.

Supplementary Table.4 Basic chemical properties of the binding between hibifolin and specific amino acids residues in SrtA.

Supplementary Table.5 Representative flavonoids and flavonol glycosides compounds with their SrtA or ClpP relative inhibition rates.

Supplementary Table.6 Comparison of inhibition rates, molecular docking data and Gaussian data of SrtA and ClpP between two pairs of representative compounds.

Supplementary Figure.1 Quality test report of hibifolin.

成都普菲德生物技术有限公司

Chengdu Pufei De Biotech Co., Ltd

Tel:86-28-82610909 Fax:86-28-81711798

Email:sc-victory@163.com 地址：成都市温江区青啤大道 319 号

棉黄素-8-O-β-D-葡糖苷酸<br>Gossypetin 8-O-beta-D-glucuronide	
检测信息<br>（Detection Information）	色谱柱（Column）：Shim-pack VP-ODS 150*4.6mm<br>流动相（Mobile phase):乙腈：0.1%磷酸水溶液=22:78<br>（Acetonitrile：0.1%phosphoric acid solution=22:78）<br>检测波长（Detection mode）：254nm<br>柱温（Column temperature）：30℃

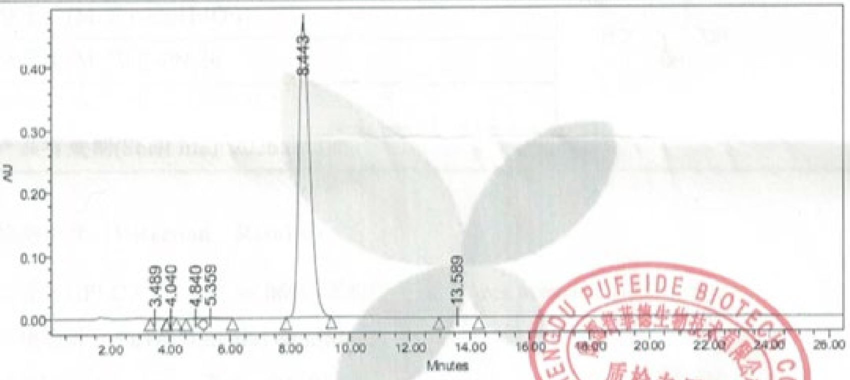

CHENGDU PUFEIDE BIOTECH CO.,LTD 成都普菲德生物技术有限公司 质检专用章

Peak Results

	Name	RT	Area	% Area
1		3.489	15871	0.13
2		4.040	14163	0.12
3		4.840	21092	0.18
4		5.359	49334	0.42
5		8.443	11725059	99.06
6		13.589	10248	0.09

Figure S1. HPLC chromatogram of hibifolin. The purity of hibifolin was 99.06%.

Supplementary Figure.2 CETSA results of purified proteins with hibifolin.

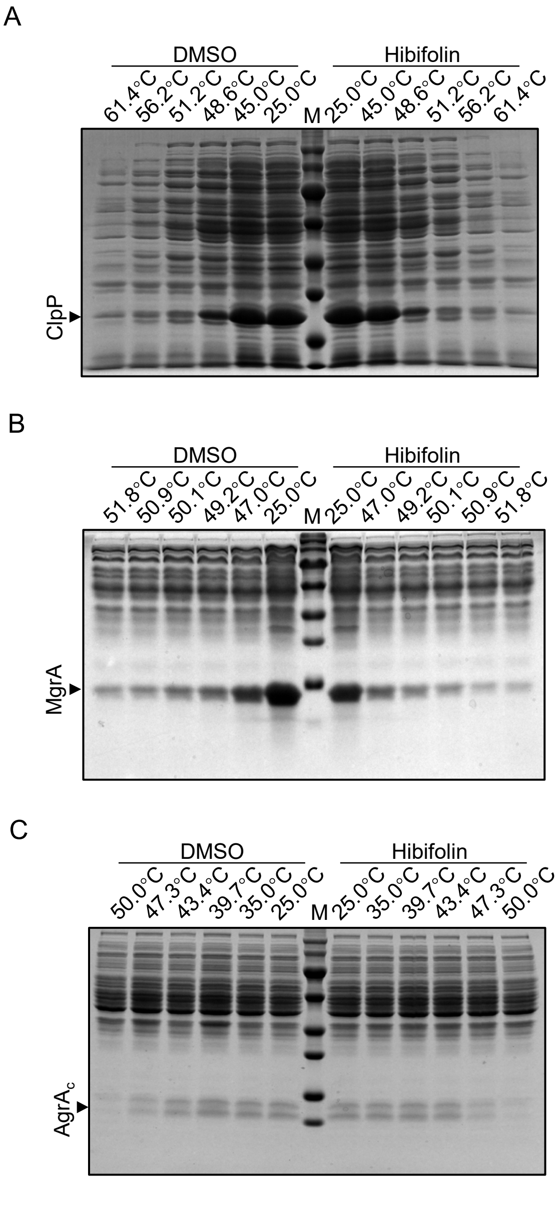

Figure S2. The full SDS-PAGE grayscale images of CETSA. (A) ClpP with or without hibifolin (128 μg/mL). (B) MgrA with or without hibifolin (128 μg/mL). (C) AgrA with or without hibifolin (128 μg/mL). Molecular weight marker shows 130, 100, 70, 50, 35, 25 ,20 and 15 kDa bands.

Table 1 Primers used in article.

Primer name	Oligonucleotide
SrtA-A104G-F	5' GGTGTAAGCTTTGGCGAAGAAAATGAATC 3'
SrtA-A104G-R	5' GATTCATTTTCTTCGCCAAAGCTTACACC 3'
SrtA-N114A-F	5' CTAGATGATCAAGCTATTTCAATTG 3'
SrtA-N114A-R	5' CAATTGAAATAGCTTGATCATCTAG 3'
SrtA-T180A-F	5' GATAAACAATTAGCCTTAATTACTTG 3'
SrtA-T180A-R	5' CAAGTAATTAAGGCTAATTGTTTATC 3'
SrtA-W194A-F	5' GAAAAGACAGGCGTTGCAGAAAAACGTAAAATC 3'
SrtA-W194A-R	5' GATTTTACGTTTTTCTGCAACGCCTGTCTTTTC 3'
SrtA-R197A-F	5' CGTTTGGGAAAAAGCCAAAATCTTTGTAGC 3'
SrtA-R197A-R	5' GCTACAAAGATTTTGGCTTTTTCCCAAACG 3'

Table 2 Antimicrobial effects of antibiotics in combination with Hibifolin in *S. aureus*.

Antibotic	MIC $_{antibiotic}$ (μg/mL)	MIC $_{combine-antibiotic}$ (μg/mL)	FIC $_{antibiotic}$	MIC $_{hibifolin}$ (μg/mL)	MIC $_{Combine-hibifolin}$ (μg/mL)	FIC $_{hibifolin}$	FICI
Cefotaxime	64	16	0.25	512	64	0.125	0.375
Doxycycline	32	16	0.5	512	64	0.125	0.625
Ceftriaxone Sodium	256	128	0.5	512	64	0.125	0.625
Vancomycin	3	3	1	512	64	0.125	1.125
Oxacillin sodium	32	32	1	512	64	0.125	1.125
Ceftiofur Sodium	32	32	1	512	64	0.125	1.125
Cefepime	64	64	1	512	64	0.125	1.125
Ceftaroline fosamil	8	16	2	512	64	0.125	2.125

Table 3 Compound library for screening SrtA inhibitors in this study.

Compound name	Relative inhibition rate (%) *	CAS
Hibifolin	65.34%	55366-56-8
Pinostrobin	/	480-37-5
2,3-Dehydrokievitone	-72.78%	74161-25-4
Bilobetin	17.81%	521-32-4
Corylifol A	36.82%	775351-88-7
5-Demethylnobiletin	54.28%	2174-59-6
5,7,8,4'-Tetramethoxyflavone	27.38%	6601-66-7
Ginkgetin	0.25%	481-46-9
Procyanidin B2	13.75%	29106-49-8
Hispidulin	50.00%	1447-88-7
Kaempferol 3,7,4'-trimethyl ether	32.57%	15486-34-7
7,4'-Dihydroxyflavone	/	2196-14-7
7,4'-Dimethoxyisoflavone	44.04%	1157-39-7
Sophoricoside	14.43%	152-95-4
Scutellarein	42.07%	529-53-3
Narcissin	44.52%	604-80-8
Tectoridin	19.98%	611-40-5
Irigenin	30.33%	548-76-5
Artemetin	32.75%	479-90-3
Sinensetin	23.78%	2306-27-6
2''-O-Galloylhyperin	42.43%	53209-27-1
Avicularin	45.33%	572-30-5
Chrysosplenetin	34.20%	603-56-5
Cosmosiin	46.75%	578-74-5
Quercetin 3-O-sophoroside	37.78%	18609-17-1
7,4'-Di-O-methylapigenin	33.49%	5128-44-9
Leucoside	27.31%	27661-51-4
Ellagic acid	/	476-66-4
Lithospermic acid	51.73%	28831-65-4
Anwulignan	15.62%	107534-93-0
Picropodophyllin	/	477-47-4
Forsythin	14.84%	487-41-2
(-)-Licarin B	38.94%	51020-87-2
Kadsurin	16.95%	51670-40-7
4′-Demethylepipodophyllotoxin	17.77%	6559-91-7
Myrislignan	1.72%	171485-39-5

* Relative inhibition of compounds at 64 μg/mL.

/ Self-fluorescence of compounds affects detection.

Table 4 Basic chemical properties of the binding between hibifolin and specific amino acids residues in SrtA.

Name	Distance	Category	Types
A:THR-180:OG - :UNN0:O	2.8	Hydrogen Bond	Conventional Hydrogen Bond
:UNN0:H - A: ASN-114:ND2	2.3	Hydrogen Bond	Conventional Hydrogen Bond
:UNN0:H - A:ALA104:O	2.1	Hydrogen Bond	Conventional Hydrogen Bond
A:ARG197:NH2 - :UNN0:O	3.2	Hydrogen Bond	Conventional Hydrogen Bond
A:TRP194:N - :UNN0:O	3.3	Hydrogen Bond	Conventional Hydrogen Bond
A:ARG197:NH1 - :UNN0	3.8	Electrostatic	Pi-Cation
A:TRP194:CD1 - :UNN0:O	3.7	Hydrogen Bond	Carbon Hydrogen Bond
A:ARG197:NH2 - :UNN0	3.8	Electrostatic	Pi-Cation

Table 5 Representative flavonoids and flavonol glycosides compounds with their SrtA or ClpP relative inhibition rates.

Compounds	Chemical structure	SrtA relative inhibition rate (%)*	ClpP relative inhibition rate (%)*	CAS	Reference
Quercetin	Flavonoids	75.01	78.92	117-39-5	[1]
Myricetin	Flavonoids	IC_{50}=48.66 μM [2]	90	529-44-2	[3]
Taxifolin	Flavonoids	56.03	17.2	480-18-2	N.P
Hinokiflavone	Flavonoids	36.16	74	19202-36-9	N.P
6,7-trimethoxyflavone	Flavonoids	16.36	55.8	572-32-7	N.P
Scutellarin	Flavonol glycosides	65.25	45.34	27740-01-8	[4]
Tamarixetin	Flavonol glycosides	26.24	73.1	603-61-2	N.P
Hibifolin	Flavonol glycosides	74.61	42.81	55366-56-8	N.P
Scutellarin Methyl Ester	Flavonol glycosides	73.81	49.2	119262-68-9	N.P
Luteolin 3′-glucuronide	Flavonol glycosides	50.46	47.1	29741-10-4	N.P
Vicenin-2	Flavonol glycosides	67.59	48.2	23666-13-9	N.P
Vincetoxicoside B	Flavonol glycosides	57.17	65.22	22007-72-3	N.P

*, Relative inhibition of compounds at 64 μg/mL.
N.P, not published.

Reference

[1] Jing S., Kong X., Wang L., Wang H., Feng J., Wei L., Meng Y., Liu C., Chang X., Qu Y., Quercetin Reduces the Virulence of S. aureus by Targeting ClpP to Protect Mice from MRSA-Induced Lethal Pneumonia, Microbiology Spectrum. (2022):e02340-02321.
[2] Hu, P., et al., Discovery of myricetin as an inhibitor against Streptococcus mutans and an anti-adhesion approach to biofilm formation. International Journal of Medical Microbiology, 2021. 311(4): p. 151512.
[3] Jing S., Wang L., Wang T., Fan L., Chen L., Xiang H., Shi Y., Wang D., Myricetin protects mice against MRSA-related lethal pneumonia by targeting ClpP, Biochemical Pharmacology. (2021) 192:114753.
[4] Wang X., Wei L., Wang L., Chen X., Kong X., Luan Y., Guan J., Guo X., Shi Y., Wang T., Scutellarin potentiates vancomycin against lethal pneumonia caused by methicillin-resistant Staphylococcus aureus through dual inhibition of sortase A and caseinolytic peptidase P, Biochemical Pharmacology. (2022) 199:114982.

Supplementary Figure.3 Interaction studies between two pairs of flavonoid and flavonoid glucosides compounds with SrtA and ClpP proteins.

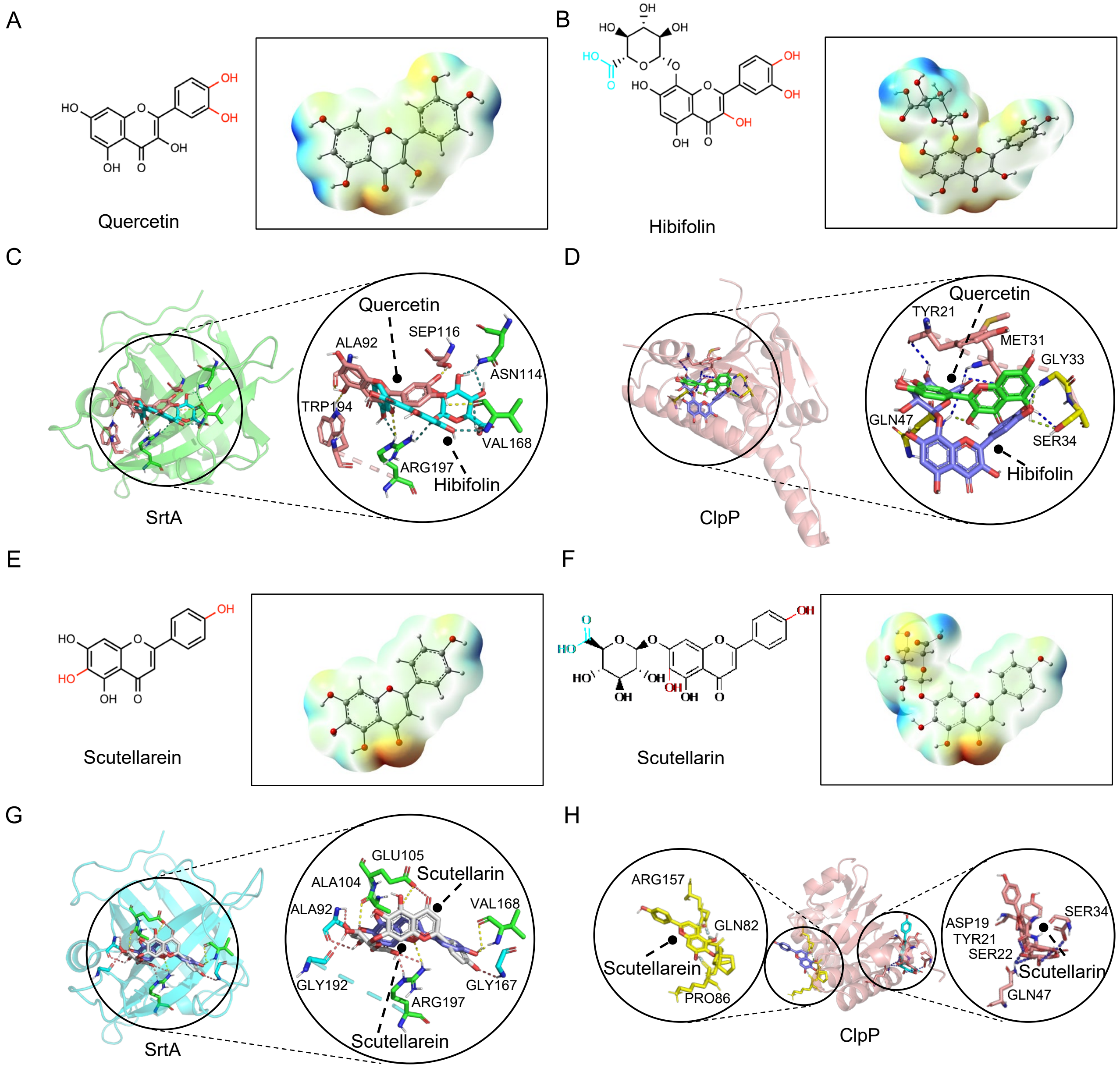

Figure S3. Chemical structures of flavonoid and flavonoid glucosides with electron density diagrams. (A) Quercetin. (B) Hibifolin. (E) Scutellarein. (F) Scutellarin. The electrostatic potential values are indicated by different colors. The red region indicates that this region is more nucleophilic; the blue region indicates that this region is more electrophilic. Molecular docking co-localization of similar ligand molecules with target proteins. (C) Molecular docking of Quercetin and Hibifolin with SrtA. (D) Molecular docking of Quercetin and Hibifolin with ClpP. (G) Molecular docking of Scutellarein and Scutellarin with SrtA. (H) Molecular docking of Scutellarein and Scutellarin with ClpP.

Table 6 Comparison of inhibition rates, molecular docking data and Gaussian data of SrtA and ClpP between two pairs of representative compounds.

Compounds	Category	CID	Mw	SrtA% 200μM	ClpP% 200μM	Gaussian ΔG (kcal/mol)**	Molecular Volume (cm^3/mol)**	Steric Hindrance Energy (kcal/mol)***	Docking Score (SrtA)****	Docking Score (ClpP)****
Quercetin	Flavonoids	5280343	302.24	75.01*	78.92	$-6.93*10^5$	194.12	62.717	-6.5	-6.9
Hibifolin	Flavonoid glucosides	5490334	494.36	74.61	42.81	$-1.17*10^6$	303.741	106.463	-7.7	-7.3
Scutellarein	Flavonoids	5281697	286.24	39.03*	N.D	$-6.46*10^5$	186.929	63.448	-6.6	-6.5
Scutellarin	Flavonoid glucosides	185617	462.36	65.25	45.34	$-1.08*10^6$	297.568	86.561	-7.8	-7.6

*, The inhibition rate of 64 μg/mL was used to estimate an inhibition rate of 200 μM. **, Calculated by Gaussian.
, Calculated by Chem 3D. *, Calculated by Autodock. N.D, not detected.
